# Acknowledgement to Reviewers of *Marine Drugs* in 2014

**DOI:** 10.3390/md13010267

**Published:** 2015-01-07

**Authors:** Marine Drugs Editorial Office

**Affiliations:** MDPI AG, Klybeckstrasse 64, CH-4057 Basel, Switzerland

The editors of *Marine Drugs* would like to express their sincere gratitude to the following reviewers for assessing manuscripts in 2014:

Aam, Berit B.
Aasen, John
Abbiati, Giorgio
Abraham, Wolf-Rainer
Acién Fernández, Francisco Gabriel
Adams, David
Agarwal, Gunjan
Agbaga, Martin-Paul
Ager, Ann
Aggelis, George
Agyei, Dominic
Ahn, Tae Seok
Aldskogius, Håkan
Alfonso, Amparo
ALMOURABIT, Ali
Alphandéry, Edouard
Altieri, Barbara
Altmann, Friedrich
Altmann, Karl-Heinz
lvarez, Mercedes
Alves, Anabela
Amat, Mercedes
Amy, Penny
Anderson, Carolyn E.
Andrade, Paula Andrade
Andrianasolo, Eric
Anraku, Makoto
Arahuetes, Rosa Mª
Arai, Masayoshi
Arakawa, Kenji
Armentano, I.
Asai, Teigo
Atluri, Venkata S.R.
Awakawa, Takayoshi
Azmi, Asfar
Bagno, Alessandro
Bahamonde, Paulina A.
Baker, Bill J.
Bakovic, Marica
Baldwin, Prof. William S.
Banack, Sandra
Banerjee, Pratik
Banskota, Arjun H.
Baptista, Mafalda S.
Barakat, Khaled
Baraldi, E.
Baran, Richard
Barbaglio, Alice
Barbosa-Pereira, Letricia
Barraud, Nicolas
Barrow, Colin
Barrow, Russel
Bates, Stephen S
Battershill, Chris
Bazhenov, Vasilii
Bazhenov, Vasily
Bazire, Alexis
Beaudry, Chris
Becker, Walter
Bedini, Emiliano
Bedoux, Gilles
Beemelmanns, Christine
Belovich, Joanne M.
Bendik, Igor
Benito, Juan M.
Bergman, Jan
Berlinck, Roberto
Berry, John
Berry, John P.
Best, Talitha
Bhatnagar, Ira
Bhattacharjya, Surajit
Bikiaris, Dimitrios
Bitzer, Jens
Blank, Lars M.
Blomme, Eric
Boddy, Christopher N.
Boeing, Wiebke J.
Bolton, Iris
Boscá, Lisardo
Botana, Luis
Botelho, Maria João
Boukouvalas, John
Bouraoui, Abderrahman
Bourdelais, Andrea
Bovee, Toine F.H.
Bowden, Bruce F
Bowers, Holly
Bowman, John P.
Bracher, Franz
Brackman, Gilles
Brahmbhatt, Tejal S.
Brandenburg, Klaus
Brayner, Roberta
Brenner, Catherine
Briasoulis, Evangelos
Brisdelli, Fabrizia
Brothers, Holly
Brück, Thomas
Brunet, Christophe
Brunner, Eike
Budge, Suzanne
Bugg, Timothy D.h.
Burnett, Karen
Burritt, David
Buttino, Isabella
Buxton, Denis
Cabral, Jaydee
Caffrey, Patrick
Caldwell, Gary
Callery, Patrick S.
Camacho, Javier
Cambedouzou, Julien
Cancela, M Leonor
Cao, Shugeng
Capon, Robert
Caputa, Michał
Carballeira, Nestor M.
Carbone, Anna
Carda, Miguel
Cardoso, Susana M.
Caretti, Fulvia
Carnevali, Oliana
Carrano, ssa Lucia
Carretti, Emiliano
Carter, Darrick
Carvajal, M.
Casanova-Nakayama, Ayako
Casely-Hayford, Maxwell
Casettari, Luca
Castell, Alina
Catchpoole, Daniel
Cavalier, Jean-François
Chakrabarti, Debopam
Chan, Andrew
Chan, Weng
Chang, Fang-Rong
Chang, Hsueh-Wei
Chaturvedula, Venkata Sai Prakash
Chen, Bing
Chen, Qiao-Hong
Chen, Qi-Yin
Chianese, Giuseppina
Chiang, Hui-Ling
Chiu, Tsai-Hsin
Cho, Yuko
Choi, Hyukjae
Choi, Jeong-Yun
Choi, Young Hae
Choi, Yung-Hyun
Chong, Parkson Lee-gau
Chou, Joshua
Christaki, Efterpi
Christian, Omar E.
Chu, Justin Jang Hann
Ciavatta, Maria Letizia
Cirés, Samuel
Clark, J. Stephen
Clive, Derrick
Coll, Josep
Colliec-Jouault, Sylvia
Colombo, Diego
Composto, Russell J.
Cooksey, Keith
Cornish, M. Lynn
Corsaro, Maria Michela
Costa, Rui R.
Costantini, Maria
Costantino, Valeria
Cox, Dermot
Crago, Jordan
Cramer, Thorsten
Cresteil, Thierry
Crimmins, Michael T.
Crupi, Pasquale
Cueto, Mercedes
Culioli, Gérald
Cutignano, Adele
Czjzek, M.
D'Acquisto, Fulvio
Dai, Yao
Dalisay, Doralyn
Dang, Max
Dang, ZhiChao
Daranas, Dr. Antonio Hernández
Dar-Fu, Prof Tai
Dash, Alekha K.
Dattelbaum, Jonathan D.
D'Auria, Maria Valeria
Davis, Andy
De Lera, Angel R.
de Morais, Rui Manuel Santos Costa
De Paolis, Michaël
De Sarro, Giovambattista
De Stefano, Daniela
Debelle, Laurent
Decoursey, Thomas
Deeds, Jonathan R.
Defoirdt, Tom
Delfourne, Evelyne
Dell'Aversano, Carmela
Deng, Hai
Derby, Charles
Desai, Umesh R.
Dharmaraj, Selvakumar
Di Cera, Enrico
Di Cosmo, Anna
Diamond, Gill
Diana, Patrizia
Dickschat, Jeroen S.
Diederich, Marc
Diez, David
Ding, Yuanqing
Dionisi, Davide
Djordjevic, Snezana
Doerksen, Robert J.
Dominguez, Herminia
Dopico-García, Sonia
Dore, Robin K
Døskeland, Stein
Dossena, Silvia
Doucette, Greg
Downey, Fred
Downing, Tim
Dozio, Elena
Dracinsky, Martin
Duarte, Ana Paula
Duca, Laurent
Dufossé, Laurent
Duncan, Katherine
DYMOCK, Brian W
Dziubla, Thomas
Easson, Cole G.
Edrada - Ebel, RuAngelie
Edwards, Christine
Edwards, Daniel J.
Egan, Suhelen
Ehrlich, Hermann
Eirín-López, José M.
Eisenreich, Wolfgang
Elaissari, Abdelhamid
Elofsson, Mikael
Ely, B.
Ernst, Robert
Esteban, Maria Ángeles
Evans, Bradley S.
Evans, Paul
Evans-Illidge, Elizabeth
Exner, Thomas E.
Faassen, Elisabeth
Farag, Ihab H.
Fassett, Robert
Fasulo, Salvatore
Fenical, William
Fernández, José J.
Ferraboschi, Patrizia
Ferreira, João Paulo
Ferreira, Marta
Fessard, Valerie
Fiedler, Hans-Peter
Firestone, Gary
Fitton, Helen
Flammang, Dr. Patrick
Florio, Tullio
Fontana, Angelo
Forino, Martino
Fox, Keith R.
Franco, Chris
Frank, Saša
Franklin, Gregory
Freitas, Ana C.
Fretz, Jackie A.
Frisvad, Jens Christian
Frontier, Alison J.
Fu, Jie
Fu, Weiqi
Fujita, Masaki
Fujita, Morifumi
Fuwa, Haruhiko
Gaboury, Louis
Gabriel, Gülsah
Gaffney, Mark
Gallo, Alessandra
Gama, Francisco
Ganesan, A.
Ganesan, Murali
Gantar, Miroslav
García Nieto, Paulino José
García, Juan
García-Caballero, Melissa
García-Gomez, Diego
Gard, Prof. Paul
Garrard, Ian
Genilloud, Olga
Geraghty, Niall W.A.
Gerbaux, Pascal
Ghavami, Saeid
Giamis, Anthony
Gill, Chris Ir
Gill, Duncan M.
Gill, Santokh
Giudici, Giovanni De
Glennon, Richard
Goldhawk, Donna E.
Gomez, Santiago
Gomez-Escribano, Juan-Pablo
Gonçalves, Olivier
Goya, Luis
Gram, Lone
Graner, Michael
Grattagliano, Ignazio
Gray, Christopher A.
Graziano, Guella
Green, Timothy J.
Grether-Beck, Susanne
Grkovic, Tanja
Gross, Harald
Gruber, Ansgar
Gruber, Christian
Gruber, Reinhard
Gu, Zhen
Guerrero, Miguel
Guihéneuf, Freddy
Guio, Joan Ramon Torrella
Gulder, Tobias
Gustafson, Kirk
Gutiérrez, Marcelino
Guzmán, Esther A.
Halaweish, Fathi
Hallegraeff, Gustaaf
Hallegraeff, Gustaaf M.
Hamilton, Gerhard
Hampel, Daniela
Han, Danxiang
Han, Seung Yun
Handy, Scott
Hanif, Novriyandi
Hannestad, Jonas
Hansen, Espen
Harada, Ken-Ichi
Harder, Jens
Hardy, John
Harinantenaina, Liva
Harvey, Brandon K.
Harwood, John
Hashimoto, Masaru
Hata, Yutaka
Hauptstein, Sabine
Hawinkels, Lukas J.a.c.
Hayashi, Yoshihiko
Hayen, Heiko
Hégarat, Le
Helquist, Paul
Hemscheidt , Thomas
Hensler, Mary E.
Heras, Angeles
Herrera-Marschitz, Mario
Herrero, Miguel
Hess, Philip
Hess, Tanja M.
Heyland, Andreas
Hidari, Kazuya I.p.j.
Hirota, Simon A.
Hoeger, Ulrich
Hoffmann, Friederike
Hogan, Deborah A.
Hollmen, Maija-Leena Kaarina
Holoshitz, Joseph
Hong, Yong-Ki
Hopper-Borge, Elizabeth A.
Hosokawa, Masashi
Hou, Ming-Hon
Houssen, Wael
Hsu, Ming-Jen
Huang, Ya-ling
Hucho, Ferdinand
Huhn, Carolin
Huigens III, Robert W.
Hukkanen, Janne
Humeida, Ute
Hussain, Rafaqat
Hwang, Seung Yong
Hyland, Niall
Ibanez, Elena
Ifuku, Kentaro
Ifuku, Shinsuke
Igarashi, Yasuhiro
Iguchi, Taisen
Iken, Katrin
Ilan, Micha
Imhoff, Johannes F.
Imperatore, Concetta
Iriti, Marcelo
Ishibashi, Masami
Ishikura, Minoru
Iwao, Masamoto
Iwao, Masatomo
Iyer, Arun
Jacobsen, Jette
Jacobson, Peer B.
Jaenicke, Elmar
Janát-Amsbury, Margit M.
Jansen, Rolf
Jarboe, Laura R.
Jensen, Paul
Jeon, You-Jin
Jesus Raposo, Maria Filomena
JIménez, Carlos
Jin, Albert
Joerger, Rolf D.
Jokela, Jouni
Jones, RL
Joo, Hong-gu
Ju, Jingfang
Jumas-Bilak, Estelle
Jung, Jee Hyung
Kadokawa, Jun-ichi
Kagami, Hideaki
Kajiya, Hiroshi
Kalinin, Vladimir
Kaloudis, Triantafyllos
Kalthoff, Holger
Kamiya, Masakatsu
Kanazawa, Kazuki
Kano, Akihiro
Kashman, Yoel
Katchik, Frederick
Kauppinen, Anu
Kawakami, Koji
Kawamura, Akira
Kawamura-Konishi, Yasuko
Kawashima, Hideki
Kearns, Philip S.
Kennedy, Jonathan
Keyzers, Rob
Khanna, Savita
Khozin-Goldberg, Inna
Kigoshi, Hideo
Kijjoa, Anake
Kikuzaki, Hiroe
Kim, Hee Sook
Kim, Hye Sun
Kim, Se-Kwon
Kim, Youngsoo
Kimber, Marc C
Kirchhofer, Daniel
Kita, Yasuyuki
Klettner, Alexa
Kluza, J.
Knölker, Hans-Joachim
Kolaczkowski, Marcin
Kolarich, Daniel
Konishi, Y
Konno, Hiroyuki
Korsnes, Mónica Suárez
Koshino, Hiroyuki
Kostin, Sawa
Krock, Bernd
Kubanek, Julia
Kucuk, Omer
Kuksis, Arnis
Kumar, Addanki P.
Kumar, Kamal
Kumirska, Jolanta
Kuo, Jimmy
Kurtboke, Ipek
Kusari, Souvik
Kuznetsov, Galina
Kwak, Jong-Young
Kwan, Jason
LaBarbera, Daniel
Labes, Antje
Lage, Olga
Lai, Daowan
Laizé, Vincent
Lam, Yulin
Landrum, John T.
Lang, Siegmund
Lanzetta, Rosa
Larcher, Gérald
Larsen, Thomas
Latini, Roberto
Latortue, Marie C.
Latz, Michael
Laurienzo, Paola
Lawrence, William S.
Lay, Jackson O.
Le Lann, Klervi
Leal, Miguel
Lebar, Matthew D.
Leblond, Jeff
Lee, Eun Yeol
Lee, Hyeyoung
Lee, Hyi-Seung
Lee, Jaehwi
Lee, Jae-Seong
Lee, Jong-Seok
Lee, Sang Kook
Lee, Tzong-shyuan
Lee, Victor
Lee, Yeon-Ju
Leflaive, Joséphine
Lembo, David
Lemmens-Gruber, Rosa
Leone, Antonella
Leu, Julie
Levitan, Orly
Lewis, Richard
Li, Jie
Li, Jun
Liang, Chia-Hua
Liao, You-di
Liermann, Johannes C.
Lim, Kwang-Hee
Lin, Yuheng
Lin, Zhenjian
Lindel, Thomas
Lindequist, Ulrike
Littlefield, Bruce A.
Liu, Chao-Lin
Liu, Delong
Liu, Zhenfeng
Lockridge, Oksana
Longatto-Filho, Adhemar
López, Jiménez
Lopez, Jose
Lopez, Pascal Jean
Lordan, Sinéad
Losso, Jack N.
Louzao, M. Carmen
Lu, Chung-kuang
Lu, Mei-Chin
Lüdeke, Steffen
Lukiw, Walter J.
Lutomski, D.
Ma, dongying
Macias Sanchez, Antonio José
Madhukar, Burra
Madihally, Sundar V.
Maeda, Hayato
Magali, Terme-Zydel
Majik, Mahesh
Maki, James
Malgesini, Beatrice
Mancini, Ines
Manger, Ron
Mangoni, Alfonso
Manzo, Emiliano
Maoka, Takashi
Mari, Frank
Marie, Benjamin
Mariussen, Espen
Martens, Dirk
Martin, Jessica
Martin, Luc J.
Martín, María Ángeles
Martin, Marjolaine
Martínez, A.
Martinez, Ana
Martins, Rosário
Mata, John Enrique
Matiadis, Dimitris
Matsugo, Seiichi
Matsumiya, Masahiro
Matsunaga, Shigeki
Matsuya, Yuji
Mazière, Cécile
Mazurak, Vera
McCall, Jennifer R.
McCarron, Pearse
McCarthy, Florence
Mccarthy, Peter
Mcdougal, Owen M.
McDougald, Diane
McHenry, Peter
McIntosh, J. Michael
Mcintyre, Lorraine
McKee, Tawnya C.
Meadus, William Jon
Mebs, Dietrich
Mechler, Adam
Medina, Miguel A´ngel
Mellon, Isabel
Mencer, Donald E
Ménez, Cécile
Menna, Marialuisa
Merel, Sylvain
Merk, Daniel
Metcalf, James
Meunier, Bernard
Meyer, Michael
Mi, Fwu-Long
Micheau, O.
Michio, Namikoshi
Miesch, Michel
Mikami, Koji
Milledge, John J.
Miller, John
Milthorpe, Bruce
Miralles-Llumà, Rosa
Moeder, Monika
Mohanam, Sanjeeva
Möhlmann, Torsten
Molgo, Jordi
Molinaro, Antonio
Molnár, Péter
Monroig, Óscar
Monti, Maria Chiara
Moomand, Khalid
Moore-Kucera, Jennifer
Moratti, S.C.
Moreno, Diego
Mori, Kenji
Morikawa, Toshio
Morimura, Shigeru
Morita, Naoki
Morris, James C.
Mossialos, Dimitris
Motti, Cherie
Mouget, Jean-Luc
Mulloy, Barbara
Munro, Murray
Murai, Toshiaki
Muramoto, Koji
Murch, Susan
Murphy, Cormac D.
Murphy, Patrick J.
Musso, Loana
Muylaert, Koenraad
Muzzarelli, Riccardo A.A.
Na, MinKyun
Naard, Adriaan J.
Nair, Satish K.
Naito, Yuji
Nájera, Carmen
Naka, Hiroaki
Nakamura, Yoji
Nakazawa, Masami
Narita, Shin-ichiro
Nasopoulou, Constantina
Nasri, Moncef
Natividad, Ruiz
Naughton, Violetta
Navarro-Vázquez, Armando
Neilan, Brett
Nekhai, Sergei
Newman, David
NG, Tzi Bun
Nicolas, Jonathan
Niedermayr, Andrea
Niedermeyer, Timo H. J.
Nielsen, Kristian Fog
Nifantiev, Nikolay
Nikolic, Dejan
Nilsson, Rune
Nishihara, Hirotomo
Nishimura, Shinichi
Nobili, Stefania
Nokami, Junzo
Nomikos, Tzortzis
Norte, Manuel
Novelli, Antonello
Nyberg, Nils T.
Oberlies, Nicholas H.
Ochi, Kozo
Oda, Tatsuya
Oh, Dong-Chan
Oh, Hyuncheol
Ohta, Shinji
O'Keefe, Barry R.
Okino, Tatsufumi
Oliveira, Ana Lígia Leandrini De
Oliveira, Paulo
Omarsdottir, Sesselja
Opatz, Till
Orecchio, Santino
Ortíz, Abel Baerga
Ortiz, Gabriela Toledo
Otsuka, Hideaki
Overkleeft, Herman S.
Overy, David
Palaniyar, Nades
Pallavicini, Alberto
Palomares, Eva Rufino
Pan, Cheol-Ho
Pan, Min-Hsiun
Papetti, Adele
Papoulias, Diana
Park, Ro-Dong
Park, Yongsoon
Patil, Jawahar
Patrauchan, Marianna A.
Pattenden, Gerald
Paul, Nicholas
Pautier, Patricia
Pawlik, Joseph R.
Pedatella, Silvana
Peigneur, Steve
Penel, N.
Penoni, Andrea
Pereira, David
Pereira, Paulo
Perrault, Louis P.
Peter Proksch, Peter
PETER, Martin
Petzelbauer, Peter
Pezzolesi, ssa Laura
Pfeifer, Blaine
Picot, Laurent
Pierce, Elizabeth
Piggott, Andrew
Pigza, Julie
Pino-Figueroa, Alejandro
Pinto, João F.
Plakas, Steve
Pluskal, Tomáš
Pohnert, Georg
Polissi, Alessandra
Polticelli, Fabiato
Polyak, Steven
Pompe, Wolfgang
Porte, Cinta
Porter, John R.
Potin, Philippe
Poynton, Helen C.
Prado, Soizic
Prince, Emily K.
Procter, David
Proksch, Peter
Puddick, Jonathan
Qiu, Xiao
Quaglia, Fabiana
Quesada, Antonio
Quinton, Loïc
Radwan, Mohamed M.
Raja, Huzefa A.
Ratledge, Colin
Rayalam, Srujana
Ré, Maria-Ines
Reglinski, Tony
Rehmann, Lars
Rehmann, Nils
Renault, Tristan
Reyes, Fernando
Rho, Jung-Rae
Rienzi, Sara Di
Rimbach, Gerald
Rittschof, Daniel
Rizvi, Syed A. A.
Rizzo, Angela Maria
Rocha-Santos, Teresa A. P.
Rodríguez, Jaime
Rohan, Lisa Cencia
Rolandi, Marco
Roldo, M.
Romano, Giovanna
Ross, Matthew
Rossius, Vassilios
Roy Chowdhury, Subir K.
Roze, Ludmila V.
Ruan, Gang
Ryu, Bomi
Ryu, Shi Yong
Sabila, Paul Sali
Saczewski, Franciszek
Sadovskaya, Irina
Sæther, Thomas
Saha, Sushanta
Saikawa, Yoko
Sainis, Ioannis
Saint Jean, Bruno
Saito, Naoki
Sakai, Ryuichi
Sakamoto, Toshio
Salim, Angela
Salomon, Christine
Salomonsson, Matilda
Samorì, Chiara
San Juan Serrano, Fuencisla
Sánchez-Moreno, Manuel
Sandjo, Louis P.
Sani, Marc-Antoine
Sanina, Nina
Sansone, Clementina
Santiago-Vazquez, Lory
Sarabia, Francisco
Sardao, Vilma
sarigiannis, Ioannis
Sato, Kenji
Sato, Shigeru
Savard, Christian
Sayanova, Olga
Scarlett, Christopher
Schenk, Peer
Schenkel, Johannes
Schievano, E.
Schmelzer, Christian E. H.
Schock, Tracey
Scholz, Bettina
Schrenk, Matt
Schröder, Heinz C.
Schulz, Stefan
Scott, Roderick
Seebeck, Florian P
Sekiguchi, Miho
Senger, Ryan S
Seo, Jeong-woo
Seubert, Erica L.
Sharma, Sushil
Shibata, Toshiyuki
Shigeno, Shuichi
Shim, Sang Hee
Shim, Young Key
Shimizu, Toshiyuki
Shin, Hee Jae
Shin, Jongheon
Shoji, Mitsuru
Shokuhfar, Tolou
Sholkamy, Eman
Shoyama, Yukihiro
Siah, Ahmed
Siddiqui, Khawar Sohail
Sieiro, Carmen
Silipo, Alba
Simpson, Tom
Siniscalco, Dario
Skretas, Georgios
Slattery, Marc
Smital, Tvrtko
Smith, Gordon
Smith, L. Courtney
Smith-Palmer, Truis
Smyth, Thomas J.
Snider, Barry
Sommer, Ulf
Son, Byeng Wha
Sone, Hideko
Song, Jinhua
Sørensen, Jens Laurids
Sosa, Anastacio Emiliano
Sousa, F. L.
Spanò, Antonio
Stanstrup, Jan
Steele, Andrew
Steiner, Andrew M.
Stensvag, Klara
Stephenson, Rachel
Stickle, Douglas
Stiger-Pouvreau, Valérie
Stoskopf, Michael
Stournaras, Christos
Suenaga, Kiyotake
Sugumaran, Manickam
Suhre, Karsten
Sumi, Shoichiro
Sung, Ping-Jyun
Sun-Ju, Kim
Surup, Frank
Süssmuth, Roderich
Sutherland, Mark
Suzuki, Tomohiko
Svoboda, Kathy
Sylvain, Rault
Tabudravu, Jioji
Tagawa, Yoh-ichi
Taglialatela-Scafati, Orazio
Takada, Kentaro
Takahashi, Daisuke
Takahashi, Koretaro
Takano, Katsura
Takeoka, Shinji
Taketani, Rodrigo Gouvêa
Tammela, Päivi
Tanaka, Junichi
Tang, Daolin
Tang, Jen-Yang
Tang, Shao-Jun
Tarapore, Rohinton S.
Tava, Aldo
Taylor, Caroline
Taylor, Erika A.
Teas, Jane
Teige, Markus
Theodorakis, Emmanuel A.
Thiruvenkatachari, Viraraghavan
Thomas, Eric
Thomas, Jim
Thomas, Olivier P.
Thomes, Paul
Thormann, Ulrich
Tidgewell, Kevin
Tinto, Winston
Tokuda, Masaharu
Torstensen, Bente E.
Tosti, Elisabetta
Touraki, Maria
Tresini, Maria
Trincone, Antonio
Tringali, Corrado
Tsigenopoulos, Costas S.
Tsodikov, Oleg
Tsodikov, Oleg V.
Tsuchikawa, Hiroshi
Tsuda, Masashi
Tsurkan, Mikhail V.
Tucker, Steven
Tulla-Puche, Judit
Tzartos, Socrates
Tziveleka, Leto-Aikaterini
Uchida, Motoharu
Uhrin, Dusan
Upton, Mathew
Usami, Yoshihide
Vale, Paulo
Valentao, Patrícia
van Altena, Ian
Van Doren, Steven R.
Van Griensven, Leo J.L.D
Van Klink, John
Van Lanen, Steven
Van Pée, Karl-heinz
Varadarajan, Sridhar
Varese, Giovanna Cristina
Varticovski, L
Varum, Kjell M.
Vasconcelos, Vitor
Vázquez, J. A.
Velu, Sadanandan E
Venditti, Alessandro
Ventrella, Vittoria
Vergara, Daniel
Vetvicka, Vaclav
Vian, Maryline Abert
Viegelmann, Christina
Vinogradov, Evgeny
Vitalone, Rocco
Vucenik, Ivana
Vuylsteke, Marnik
Wachi, Masaaki
Wagoner, Ryan
Wakimoto, Toshiyuki
Wang, Chenguang
Wang, Hongbing
Wang, Zheng
Wary, Kishore K.
Watanabe, Ryuichi
Wegscheid, Claudia
Wei, Mei
Welker, Martin
Weng, Han-Rong
West, Lyndon M.
Westrick, Judy
White, Alex
White, Robert L.
Wickramasinghe, Wasantha
Williams, Becky
Williams, Paul
Williams, Philip
Williams, Robert M.
Winkler, Jeffrey D.
Winlow, Bill
Winter, Esti
Winter, Josef
Wisniewski-Dyé, Florence
Witulski, Bernhard
Wojtovich, Andrew
Wolfe, Gordon
Wolz, Christiane
Wong, Jack Ho
Wösten, Han A. B.
Wright, Amy
Wright, Anthony
Wu, Chang-jer
Wu, Qingli
Wu, Shih-Hsiung
Wu, Yu-Jen
Xia, Menghang
Yakes, Betsy
Yamada, Takeshi
Yamaguchi, M.
Yamaguchi, Takumi
Yamaguchi, Yoshiki
Yamasaki, Takahiro
Yamashita, Mari Yotsu
Yan, Jun
Yan, Ning
Yang, Da-Qing
Yang, Ji Won
Yang, Jiong
Yazdanparast, Razieh
Ye, Tao
Yih, Ling-huei
Yoon, Kyung Byung
Yoon, Moon-Young
Yoshida, Masahiro
Yu, Richard
Zahler, Stefan
Zampella, Angela
Zan, Jindong
Zauner, Stefan
Zeng, Huaqiang
Zeng, Jia
Zenger, Kyall R
Zengler, Karsten
Zheng, Qi-Huang
Zhu, Xuegong
Zibaee, Arash
Zimba, Paul V.
Zorzano, Antonio
Zubía, Eva
Zuck, Karina

